# Supplementary material for: Subtilisin-Involved Morphology Engineering for Improved Antibiotic Production in Actinomycetes
Source: Biomolecules. 2020 Jun 3;10(6):851. doi: 10.3390/biom10060851 (PMC7356834; doi:10.3390/biom10060851)
Supplement: Supplementary file 1 [file biomolecules-10-00851-s001.pdf]

biomolecules

Article

Subtilisin-Involved Morphology Engineering for Improved Antibiotic Production in Actinomycetes

Yuanting Wu [1,2], Qianjin Kang [1,2], Li-Li Zhang [3] and Linquan Bai [1,2,3,*]

1 State Key Laboratory of Microbial Metabolism, School of Life Sciences & Biotechnology, Shanghai Jiao Tong University, Shanghai 200204, China; yuantingwu1987@sjtu.edu.cn (Y.W.); qjkang@sjtu.edu.cn (Q.K.)
2 Joint International Research Laboratory of Metabolic & Developmental Sciences, Shanghai Jiao Tong University, Shanghai 200240, China
3 College of Life Science, Tarim University, Alar 843300, China; zhang63lyly@sina.com
* Correspondence: bailq@sjtu.edu.cn

Table S1. Strains and plasmids used in this study.

Strains or Plasmids	Features	Sources
	A. pretiosum	
ATCC 31280	Wild-type producer for ansamitocins	ATCC
WYT-1	Deletion of *APASM_4714* in *A. pretiosum* ATCC 31280	This work
WYT-2	Deletion of *APASM_1687* in *A. pretiosum* ATCC 31280	This work
WYT-3	Deletion of *APASM_4313* in *A. pretiosum* ATCC 31280	This work
WYT-4	Deletion of *APASM_1806* in *A. pretiosum* ATCC 31280	This work
WYT-5	Deletion of *APASM_4178* in *A. pretiosum* ATCC 31280	This work
WYT-6	Deletion of *APASM_4084* in *A. pretiosum* ATCC 31280	This work
WYT-7	Deletion of *APASM_4728* in *A. pretiosum* ATCC 31280	This work
WYT-8	Deletion of *APASM_3971* in *A. pretiosum* ATCC 31280	This work
WYT-9	Deletion of *APASM_4527* in *A. pretiosum* ATCC 31280	This work
WYT-10	Deletion of *APASM_1927* in *A. pretiosum* ATCC 31280	This work
WYT-11	Deletion of *APASM_3372* in *A. pretiosum* ATCC 31280	This work
WYT-12	Deletion of *APASM_2967* in *A. pretiosum* ATCC 31280	This work
WYT-13	WYT-5 complemented with cloned *APASM_4178* under the control of *kasOp** promoter, Apr^R	This work
WYT-15	Deletion of *APASM_1021* in *A. pretiosum ATCC 31280*	This work
WYT-16	Deletion of *APASM_3332* in *A. pretiosum ATCC 31280*	This work
WYT-17	Deletion of *APASM_4306* in *A. pretiosum ATCC 31280*	This work
WYT-18	Deletion of *APASM_5462* in *A. pretiosum ATCC 31280*	This work
WYT-20	*A. pretiosum* ATCC 31280 with cloned *APASM_3064* under the control of *kasOp** promoter, Apr^R	This work
WYT-21	*A. pretiosum* ATCC 31280 with cloned *APASM_6209* under the control of *kasOp** promoter, Apr^R	This work
WYT-24	*A. pretiosum* ATCC 31280 with vector plasmid pLQ856, Apr^R	This work
WYT-25	WYT-15 with vector plasmid pLQ856, Apr^R	This work
	Streptomyces albus	
BK 3-25	High-yield producer of salinomycin	[1]
WYT-26	BK 3-25 with vector plasmid pLQ856, Apr^R	This work
WYT-27	BK 3-25 with cloned *APASM_4178* under the control of *kasOp** promoter, Apr^R	This work
	Streptomyces hygroscopicus	
TL01	High-yield producer of validamycin	[2]
WYT-28	TL01 with vector plamid pPM927, Thio^R	This work
WYT-29	TL01 with cloned *APASM_4178* under the control of *kasOp** promoter, Apr^R	This work
	E. coli	
DH10B	F$^-$ *mcr*A Δ(*mrr-hsd*RMS-*mcr*BC) ϕ80*lac*ZΔM15 Δ*lac*X74 *rec*A1 *end*A1 *ara*D139 Δ(*ara, leu*)7997 *gal*U *gal*K λ$^-$ *rsp*L *nup*G	Invitrogen
ET12567(pUZ8002)	F$^-$ *dam*-13::Tn9 *dcm*-6 *hsd*M *hsd*R *zjj-202*::Tn10 *rec*F143 *gal*K2 *gal*T22 *ara*-14 *lac*Y1 *xyl*-5 *leu*B6 *thi*-1 *ton*A31 *rps*L136 *his*G4 *tsx*-78 *mtl*-1 *gln*V44 (Cml^R Kan^R)	[3]
BL21(DE3)/pLysE	F$^-$ *ompT* *hsd*S$_B$(r$_B^-$ m$_B^-$) *gal dcm* (DE3) pLysE (Cml^R)	[4]

Table S1. *Cont.*

Strains or Plasmids	Features	Sources
	Plasmids	
pBluescript KS-	*bla*, *lac*Z, *ori*F1	Stratagene
pIB139	*attP*, *int*, *ori*T, *PermE**, *aac(3)IV*	[5]
pJTU1278	*rep*-pIJ101, *bla*, *tsr*, *ori*T	[6]
pET28a	*rep*-pBR322, *pT7*, His6-tag, *neo*	Novogen
pPM927	pSAM2 derivative, *tsr*, *ori*T	[7]
pLQ855	Construct for the deletion of *APASM_4178*	This work
pLQ856	pSET152 with promoter *kasOp**	This work
pLQ864	Construct for the deletion of *APASM_1021*	This work
pLQ869	pLQ856 with cloned *APASM_3064* under the control of *kasOp**	This work
pLQ870	pLQ856 with cloned *APASM_6209* under the control of *kasOp**	This work
pLQ874	pLQ856 with cloned *APASM_4178* under the control of *kasOp**	This work
pLQ881	pET28a with cloned *APASM_1021* for the overexpression of APASM_1021	This work
pLQ893	pPM927 with cloned *APASM_4178* under the control of *kasOp**	This work

Table S2. Primers used in this study.

Primers	Sequence (5′-3′)
4714-L-F	ATAGGATCCCAGCGACAGCTCGAACAG (*Bam*HI), for *APASM_4714* deletion
4714-L-R	ATAGAATTCGGTCCTCGGTGGTCATGT (*Eco*RI), for *APASM_4714* deletion
4714-R-F	ATAGAATTCACAACGACCGCACCAGGA (*Eco*RI), for *APASM_4714* deletion
4714-R-R	ATAAAGCTTATGATCTTGCCGCCGTACAG (*Hin*dIII), for *APASM_4714* deletion
1687-L-F	ATAGGATCCGCCAGCTCCAGGTTCTCC (*Bam*HI), for *APASM_1687* deletion
1687-L-R	ATAGAATTCGTGATCGTGTTCCTCCAGTTC (*Eco*RI), for *APASM_1687* deletion
1687-R-F	ATAGAATTCGCCCGCCATCACGAACTC (*Eco*RI), for *APASM_1687* deletion
1687-R-R	ATAAAGCTTTGCCACCACCACGTCCTC (*Hin*dIII), for *APASM_1687* deletion
4313-L-F	ATAGGATCCCGAGAACGGCACGACCAG (*Bam*HI), for *APASM_4313* deletion
4313-L-R	ATAGAATTCCGATCTCCTCCTCGGTGAAC (*Eco*RI), for *APASM_4313* deletion
4313-R-F	ATAGAATTCTGGTGTTCGTGGTGGTGAA (*Eco*RI), for *APASM_4313* deletion
4313-R-R	ATAAAGCTTCTGGTGCGGGTAGGAGTC (*Hin*dIII), for *APASM_4313* deletion
1806-L-F	ATAGGATCCATCCTGGTCCGCGAGGAG (*Bam*HI), for *APASM_1806* deletion
1806-L-R	ATAGAATTCCGACCCGCTGTCCTGATC (*Eco*RI), for *APASM_1806* deletion
1806-R-F	ATAGAATTCACTGTGTCGCAACCTCTCC (*Eco*RI), for *APASM_1806* deletion
1806-R-R	ATAAAGCTTGACCCGATGCCGATGATGA (*Hin*dIII), for *APASM_1806* deletion
4178-L-F	ATATCTAGAGTCCGGGTGCTGCTGGGCC (*Xba*I), for *APASM_4178* deletion
4178-L-R	ATAGAATTCTCGTCCACTTCGGACTCGTC (*Eco*RI), for *APASM_4178* deletion
4178-R-F	ATAGAATTCACCACCCCGGCGGAGAGC (*Eco*RI), for *APASM_4178* deletion
4178-R-R	ATAAAGCTTCCAACGGCGAGTACTACTT (*Hin*dIII), for *APASM_4178* deletion
4084-L-F	ATATCTAGAGTAGCGGAGGACCACCGT (*Xba*I), for *APASM_4084* deletion
4084-L-R	ATAGAATTCGCATACGCCTGCCGAACC (*Eco*RI), for *APASM_4084* deletion
4084-R-F	ATAGAATTCGACGCAGGTGCTGTCCTT (*Eco*RI), for *APASM_4084* deletion
4084-R-R	ATAAAGCTTCCGTCGCCAGCTACATCA (*Hin*dIII), for *APASM_4084* deletion
4728-L-F	ATATCTAGAGCAGAGCGGTGACGAGTC (*Xba*I), for *APASM_4728* deletion
4728-L-R	ATAGAATTCCGTTGCAGCTCAGGGTGTT (*Eco*RI), for *APASM_4728* deletion
4728-R-F	ATAGAATTCAGAAGTCCCTGCGGGCCTG (*Eco*RI), for *APASM_4728* deletion
4728-R-R	ATAAAGCTTGGCATGAGGTGGTCGAACAG (*Hin*dIII), for *APASM_4728* deletion
3971-L-F	ATATCTAGAGGACCTTCGACGACGAGG (*Xba*I), for *APASM_3971* deletion
3971-L-R	ATAGAATTCGAGCTGGCCTAGGTGCTG (*Eco*RI), for *APASM_3971* deletion
3971-R-F	ATAGAATTCCAGCCGTCGCTCCACCAG (*Eco*RI), for *APASM_3971* deletion
3971-R-R	ATAAAGCTTGTCGTGCTCCGCCGCTAC (*Hin*dIII), for *APASM_3971* deletion
4527-L-F	ATATCTAGACGGCGATGATCCAGGAGTC (*Xba*I), for *APASM_4527* deletion
4527-L-R	ATAGAATTCGTACGACGACGGCAGGAC (*Eco*RI), for *APASM_4527* deletion
4527-R-F	ATAGAATTCACCACGACCGACTGGGAG (*Eco*RI), for *APASM_4527* deletion
4527-R-R	ATAAAGCTTGTACCGCTACCGCTACGAC (*Hin*dIII), for *APASM_4527* deletion
1927-L-F	ATATCTAGATACGGCTTCCACGGGCTGT (*Xba*I), for *APASM_1927* deletion
1927-L-R	ATAGAATTCCGACAGAGACAGGGACACAAC (*Eco*RI), for *APASM_1927* deletion
1927-R-F	ATAGAATTCGATGGCGCTGCGCGGTTT (*Eco*RI), for *APASM_1927* deletion
1927-R-R	ATAAAGCTTGCGTTCGGATTCGGCACTTC (*Hin*dIII), for *APASM_1927* deletion
3372-L-F	ATATCTAGAGAAGGCGAGTTCTGGTTGTG (*Xba*I), for *APASM_3372* deletion
3372-L-R	ATAGAATTCGGCGTGCAGGAAGGTGAT (*Eco*RI), for *APASM_3372* deletion

Table S2. *Cont.*

Primers	Sequence (5′-3′)
3372-R-F	ATAGAATTCCCACGTCCACGGCTACCT (*Eco*RI), for *APASM_3372* deletion
3372-R-R	ATAAAGCTTTGTGGTGGTGGCGTTCCT (*Hin*dIII), for *APASM_3372* deletion
2967-L-F	ATATCTAGATGTGGTGGTGGCGTTCCT (*Xba*I), for *APASM_2967* deletion
2967-L-R	ATAGAATTCTGACCACGGCGATGTCCAT (*Eco*RI), for *APASM_2967* deletion
2967-R-F	ATAGAATTCAGTCGGACTCGCACAATC (*Eco*RI), for *APASM_2967* deletion
2967-R-R	ATAAAGCTTGTTCGCGCAGATGATGCT (*Hin*dIII), for *APASM_2967* deletion
4178-F	ATACATATGCCTCTCCGGAAAGCGCTCTCCG (*Nde*I)
4178-R	ATAGAATTCTCAGCGCAGCGCGTACGCCCGGAT (*Eco*RI)
4714-ver-F	CGTTCGTCCTGGTCTTCA
4714-ver-R	ACTGCGGCAGGTAGATCA
1687-ver-F	GCGAAGCACAGGTACTTC
1687-ver-R	GGTGGCGTGGAACTACT
4313-ver-F	GGTGACCCGCAGCATCAT
4313-ver-R	GTCGGTGCCGAACCAGTG
1806-ver-F	TACTCGGTGTACGGCGTC
1806-ver-R	TTCCCAGTGGTATTTCCCT
4178-ver-F	GTACGCCCGGATAATGGT
4178-ver-R	GAGCGGAGGAGAACACAT
4084-ver-F	ACATCCGACACCAATCAC
4084-ver-R	AGCATCTGTTCTCCTTTCTC
4728-ver-F	CTGGAAGCCGACCGAGAC
4728-ver-R	CGTGGGAGAGCAGGAGGA
3971-ver-F	CTACCACGGGCACTTCAA
3971-ver-R	TCAACGACTCCACCTACG
4527-ver-F	GGTAGACCAGGACCATGCC
4527-ver-R	CCGTGACGATGGACGACTC
1927-ver-F	GTGAACCGCCAGCTCAGG
1927-ver-R	GTGAACCGCCAGCTCAGG
3372-ver-F	CCGAGGACCAGCAGCAGTT
3372-ver-R	TCGTCGAGCACCAGCACCA
2967-ver-F	GTCTTCTACGGCATCACGAC
2967-ver-R	CGCTGGAGGTGTACGAGG
1021-L-F	ATAGGATCCGTAGTCGCCGAAGCCGTC (*Bam*HI)
1021-L-R	ATAGAATTCGGTAGAGCCTCCGGTCCG (*Eco*RI)
1021-R-F	ATAGAATTCGCTGCTGGAGACCACGGA (*Eco*RI)
1021-R-R	ATAAAGCTTGCAGCACACCAGCCAGTC (*Hin*dIII)
1021-ver-F	GTAGAGCAGCAGGTTCAC
1021-ver-R	CAAGCAAGGAGCCTTTCG
4178-RT-F	CCTGCTCGTAGACGGTCG
4178-RT-R	TGTCCGGGACCTCGATGG
P4178-F-FAM	AGGGGAACGGGGGCGGGGT
P4178-R	TGGAGCCGCTGACGGGGA
3064-F	ATACATATGGTGCGGAACCACGGAGTGAAAGGGA (*Nde*I)
3064-R	ATAGAATTCTCAGGCGGGCTGTGACGCCTCCAC (*Eco*RI)
6209-F	ATACATATGAATCCCATGACACCTATTC (*Nde*I)
6209-R	ATAGAATTCTCAGTCCTTGGCGAACGAGAACAC (*Eco*RI)
1021-F	ATA CATATGGTGGTCGTGCTCGTGCTGCCGGACGT (*Nde*I)
1021-R	ATA GAATTCTCACCCGGCGGAGCGGAACGT (*Eco*RI)

Table S3. Compositions of five media used for morphology observation.

Chemicals	Concentration (g/L)
Medium-1 seed medium	
Yeast extract	4.0
Malt extract	10.0
Glucose	4.0
Medium-1 Fermentation medium	
Glucose	60.0
Maltose	30.0
Cotton seed meal	5.3
Yeast extract	4.5
K_2HPO_4	0.5
$MgSO_4{\cdot}7H_2O$	0.002
$CaCO_3$	5.0
L-Valine	0.3%(*w*/*v*)
Medium-2 seed medium	
Soluble starch	30.0
Soybean flour	10.0
Glucose	20.0
Corn steep liquor powder	10.0
Tryptone soya broth	5.0
NaCl	0.3
$CaCO_3$	5.0
Medium-2 Fermentation medium	
Glucose	20.0
Corn starch	30.0
Cotton seed meal	30.0
$CaCl_2$	10.0
$CaCO_3$	5.0
Isobutanol	54 mM
Medium-3 seed medium	
Tryptone soya broth	30.0
Yeast extract	5.0
Sucrose	103.0
Medium-3 Fermentation medium	
Yeast extract	4.0
Malt extract	10.0
Glucose	4.0
Medium-4 seed medium	
Tryptone soya broth	30.0
Yeast extract	5.0
Sucrose	103.0
Medium-4 Fermentation medium	
Yeast extract	4.0
Malt extract	10.0
Glucose	4.0
Isobutanol	36 mM
Medium-5 seed medium	
Glycerol	10.0
Yeast extract	10.0
Glucose	5.0
Beef extract	10.0
NaCl	3.0
Medium-5 Fermentation medium	
Glucose	5.0
$FeSO_4{\cdot}7H_2O$	0.002
$MgSO_4{\cdot}7H_2O$	0.49
K_2HPO_4	0.5
$CaCO_3$	2.0
Yeast extract	10.0
Glycerol	40.0
Sucrose	2.5

Table S4. Candidate genes involved in mycelial fragmentation according to RNA-seq analysis.

Genes	Annotation	Transcription*		
		**15 h/*hrdB***	**18 h/*hrdB***	**24 h/*hrdB***
APASM_1687	urea ABC transporter	0.43	0.06	133.23
APASM_1806	cyclase dehydratase	168.38	28.66	395.41
APSAM_1927	protein kinase	12.44	3.04	21.96
APASM_2967	hypothetic protein	0.32	0.07	0.34
APASM_3372	xylulose 5-phosphate phosphoketolase	2.77	0.63	10.79
APASM_3971	α-mannosidase	1.07	0.24	1.70
APASM_4084	transcriptional regulator	1.43	0.26	2.90
APASM_4178	peptidase S8 and S53 subtilisin	0.74	0.12	0.48
APSAM_4313	ABC transporter	0.62	0.10	0.62
APASM_4527	RHS family protein	0.72	0.17	0.57
APASM_4714	collagen like surface protein	0.68	0.08	1.09
APASM_4728	ABC transporter	0.43	0.10	0.69

* Mycelia were collected at 15, 18 and 24 h of the fermentation for RNA-seq analysis.

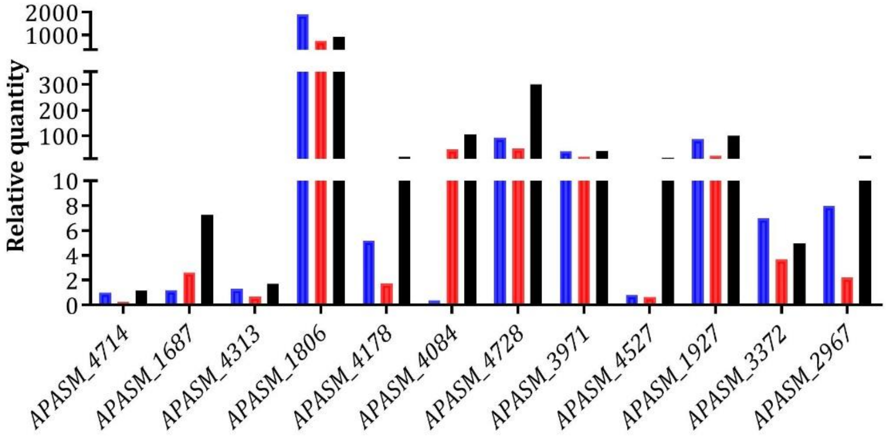

Figure S1. qRT-PCR verification of the transcription of genes selected by RNA-seq analysis. Transcription levels at 15, 18 and 24 h are shown in blue, red and black, respectively.

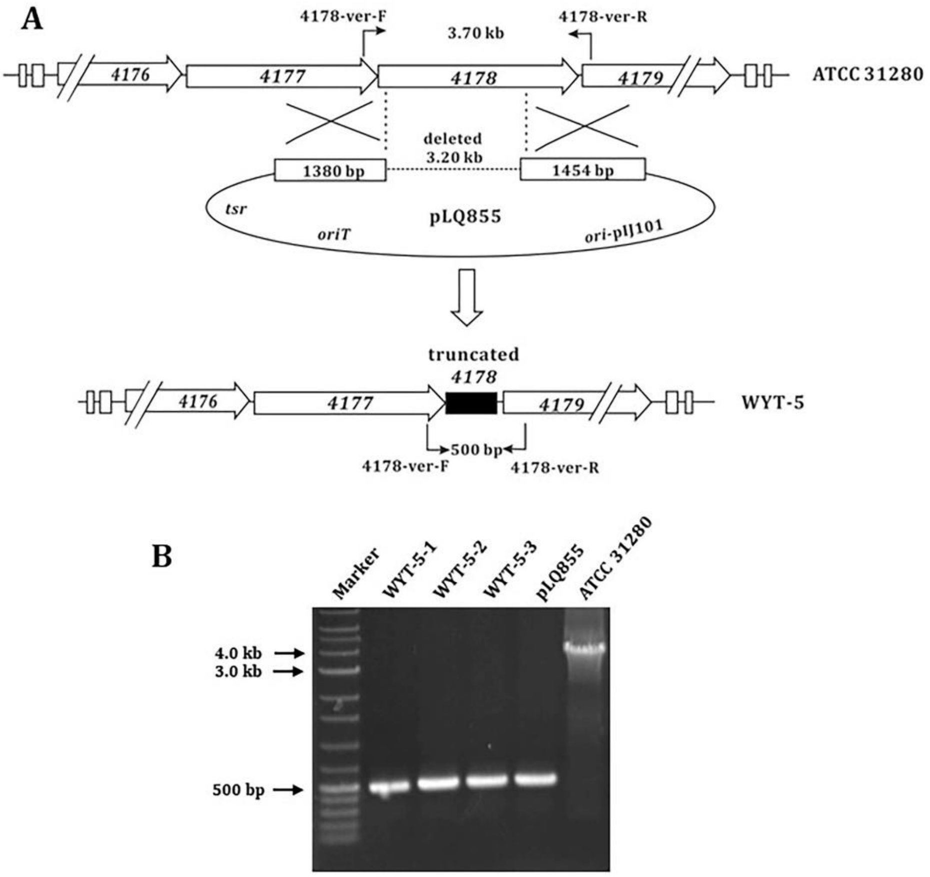

Figure S2. Deletion of gene *APASM_4178* in *A. pretiosum* ATCC 31280. (**A**) Schematic construction of Δ*APASM_4178* mutant WYT-5. 4178-ver-F/R are primers used for the verification of gene deletion by PCR. (**B**) Gel electrophoresis of the PCR-amplified fragments using total DNAs of *A. pretiosum* ATCC 31280 or WYT-5 as templates and primers 4178-ver-F/R.

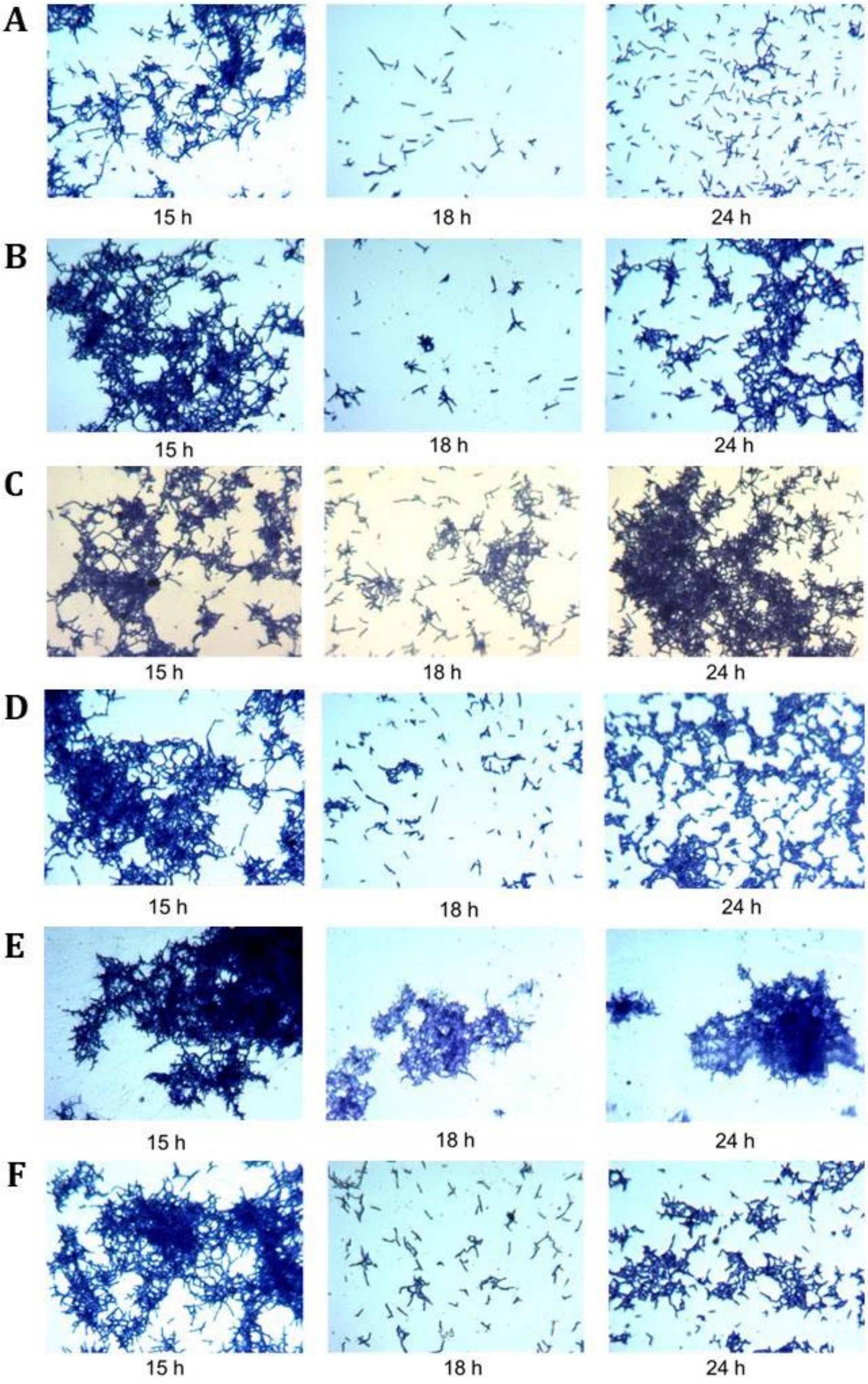

Figure S3. *Cont.*

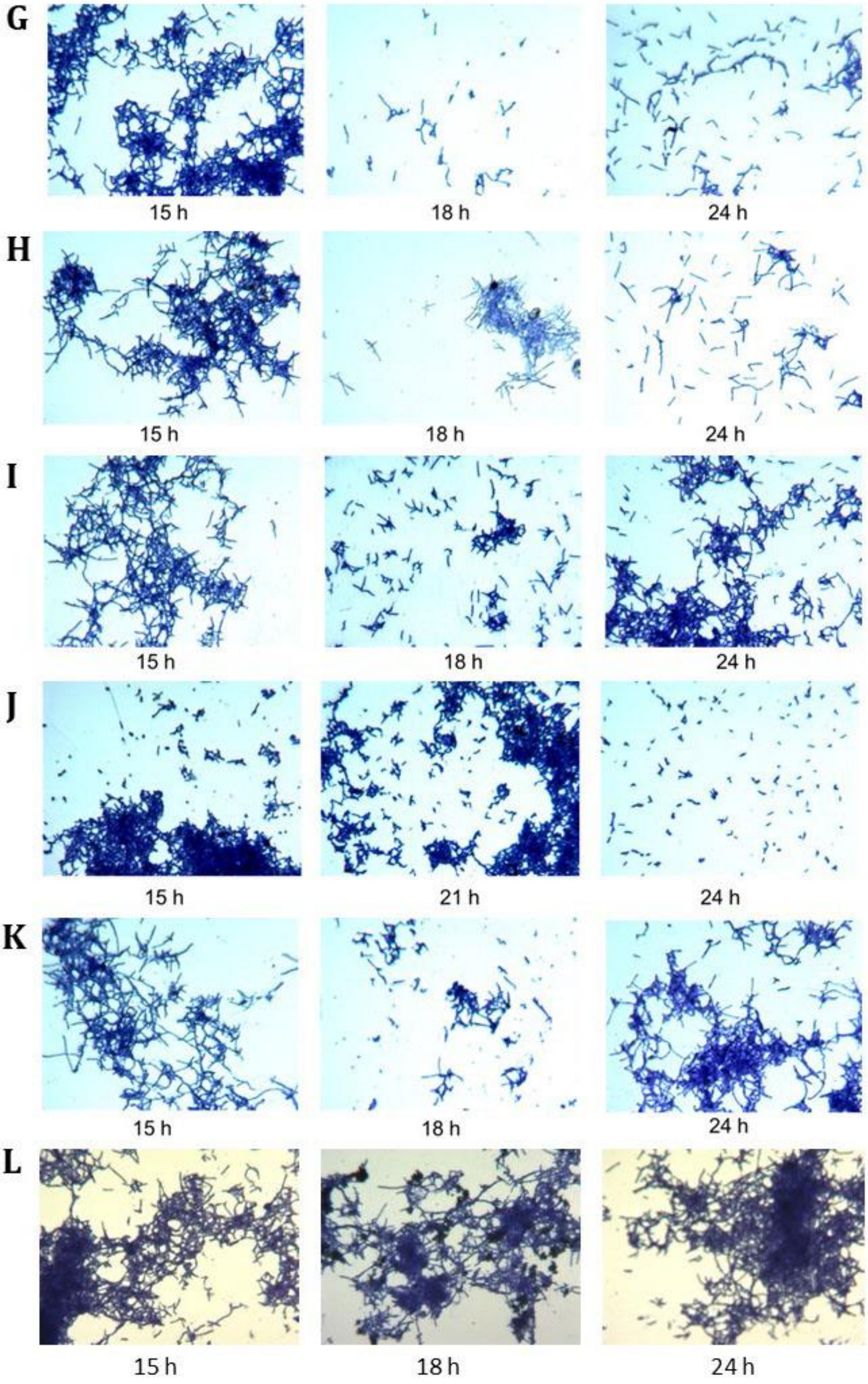

Figure S3. Mycelial morphology of mutants of 12 genes selected by RNA-seq analysis. (**A**) Δ*APASM_4714*. (**B**) Δ*APASM_1687*. (**C**) Δ*APASM_4313*. (**D**) Δ*APASM_1806*. (**E**) Δ*APASM_4084*. (**F**) Δ*APASM_4728*. (**G**) Δ*APASM_3971*. (**H**) Δ*APASM_4527*. (**I**) Δ*APASM_1927*. (**J**) Δ*APASM_3372*. (**K**) Δ*APASM_2967*. (**L**) Δ*APASM_4178*.

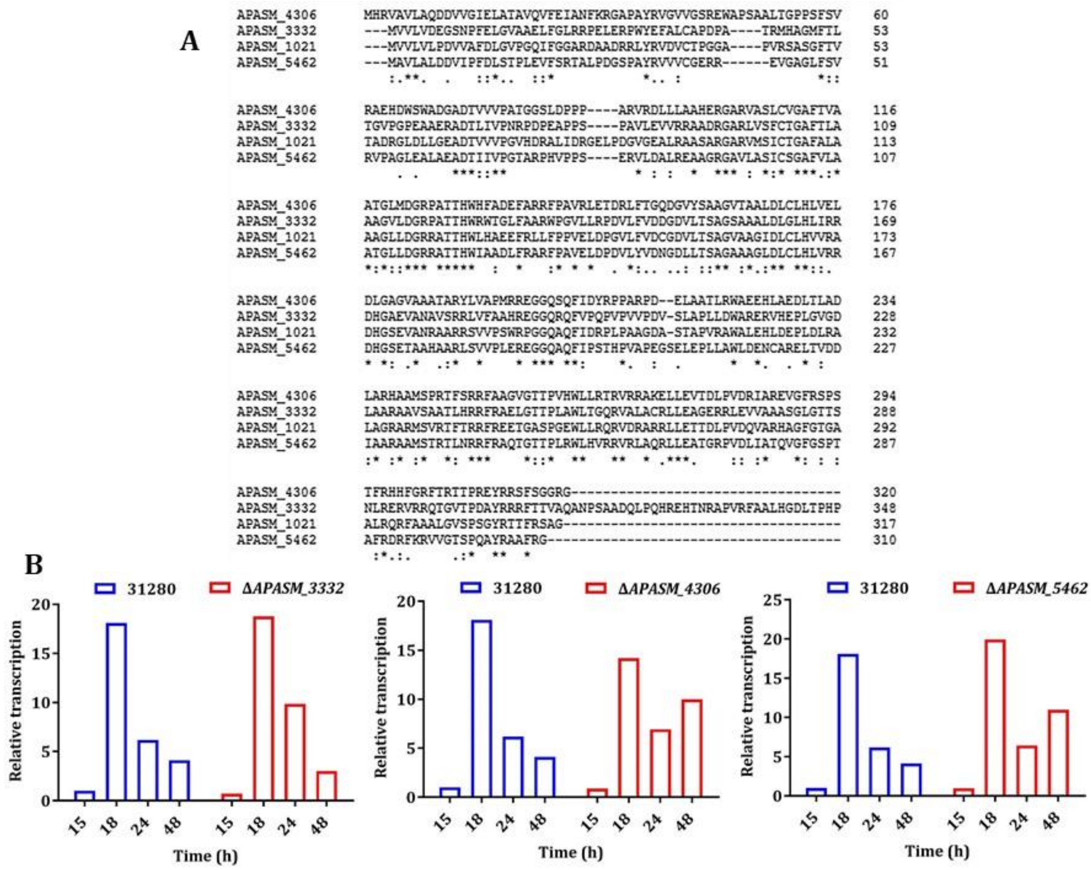

Figure S4. Sequence alignments of four AdpA-like proteins and effects of their deletions on the transcription of *APASM_4178*. (**A**) Sequence alignments of four AdpA-like proteins from *A. pretiosum* ATCC 31280 using Clustal Omega [8]. (**B**) Effect of individual deletion of three AdpA-like-coding genes on the transcription of *APASM_4178*. .

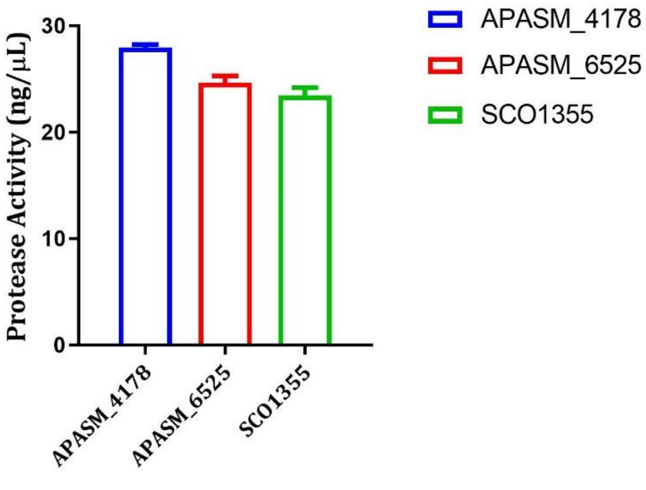

Figure S5. Peptidase activities of APASM_4178, APASM_6525, and SCO1355. APASM_6525, a 519-aa subtilisin-like serine peptidase from *A. pretiosum* ATCC 31280. SCO1355, a 537-aa subtilisin-like serine peptidase from *Streptomyces coelicolor* A3(2) [9]. Peptidase activity was measured using protease assay kit (BBI 786-028). Fluorescent protein substrate was diluted to approximate concentrations before

analysis, and samples were measured at 570 nm after incubation to determine peptidase activity. Chemically stabilized trypsin was serially diluted from 20 to 1.25 ng/μL and reacted with 2.5 μL substrate, serving as a general peptidase standard.

References

1. Zhang, X.; Lu, C.; Bai, L. Mechanism of salinomycin overproduction in *Streptomyces albus* as revealed by comparative functional genomics. *Appl. Microbiol. Biot.* **2017**, *101*, 4635–4644.
2. Tan, G.-Y.; Bai, L.; Zhong, J.-J. Exogenous 1,4-butyrolactone stimulates A-factor-like cascade and validamycin biosynthesis in *Streptomyces hygroscopicus* 5008. *Biotechnol. Bioeng.* **2013**, *110*, 2984–2993.
3. Paget, M.S.; Chamberlin, L.; Atrih, A.; Foster, S.J.; Buttner, M.J. Evidence that the extracytoplasmic function sigma factor σ^E is required for normal cell wall structure in *Streptomyces coelicolor* A3(2). *J. Bacteriol.* **1999**, *181*, 204–211.
4. Moffatt, B.A.; Studier, F.W. T7 lysozyme inhibits transcription by T7 RNA polymerase. *Cell* **1987**, *49*, 221–227.
5. Wilkinson, C.J.; Hughes-Thomas, Z.A.; Martin, C.J.; Bohm, I.; Mironenko, T.; Deacon, M.; Wheatcroft, M.; Wirtz, G.; Staunton, J.; Leadlay, P.F. Increasing the efficiency of heterologous promoters in actinomycetes. *J. Mol. Microb. Biotech.* **2002**, *4*, 417–426.
6. He, Y.; Wang, Z.; Bai, L.; Liang, J.; Zhou, X.; Deng, Z. Two pHZ1358-derivative vectors for efficient gene knockout in *Streptomyces*. *J. Microbiol. Biotechn.* **2010,** *20*, 678–682.
7. Smokvina, T.; Mazodier, P.; Boccard, F.; Thompson, C.J.; Guerineau, M. Construction of a series of pSAM2-based integrative vectors for use in actinomycetes. *Gene* **1990**, *94*, 53–59.
8. Sievers, F.; Wilm, A.; Dineen, D.; Gibson, T.J.; Karplus, K.; Li, W.; Lopez, R.; McWilliam, H.; Remmert, M.; Söding, J.; et al. Fast, scalable generation of high-quality protein multiple sequence alignments using Clustal Omega. *Mol. Syst. Biol.* **2011**, *7*, 539.
9. Kim, D.W.; Hesketh, A.; Kim, E.S.; Song, J.Y.; Lee, D.H.; Kim, I.S.; Chater, K.F.; Lee, K.J. Complex extracellular interactions of proteases and a protease inhibitor influence multicellular development of *Streptomyces coelicolor*. *Mol. Microbiol.* **2008**, *70*, 1180–1193.

© 2020 by the authors. Licensee MDPI, Basel, Switzerland. This article is an open access article distributed under the terms and conditions of the Creative Commons Attribution (CC BY) license (http://creativecommons.org/licenses/by/4.0/).
